# Childhood Trauma Exposure Associated With a More Severe Course of Metabolic Disturbances in Bipolar Disorder: A 36‐Month Longitudinal Prospective Cohort Study

**DOI:** 10.1002/brb3.71789

**Published:** 2026-10-24

**Authors:** Juan F. Galvez‐Florez, Hernán F. Guillen‐Burgos, Sergio Moreno‐López, Mariana Ramírez‐Posada, Juan‐Manuel Anaya, Roger S. McIntyre

**Affiliations:** ^1^ Clínica De Nuestra Señora De La Paz Orden Hospitalaria de San Juan De Dios, Center for Clinical and Translational Research Bogotá D.C. Colombia; ^2^ Center For Clinical and Translational Research Universidad Simón Bolívar Barranquilla Colombia; ^3^ Department of Psychiatry and Mental Health Pontificia Universidad Javeriana Bogotá D.C. Colombia; ^4^ School of Medicine Universidad De Los Andes Bogotá D.C. Colombia; ^5^ Department of Dermatology Stanford University Palo Alto California USA; ^6^ Healthy Longevity Center Universidad de la Costa Barranquilla Colombia; ^7^ Department of Psychiatry University of Toronto Toronto Canada

**Keywords:** bipolar disorder, body mass index, BMI, childhood trauma, HbA1c, high‐sensitivity C‐reactive protein, metabolic outcomes

## Abstract

**Background:**

Childhood trauma (CT) is a recognized risk factor for adverse psychiatric and physical health outcomes in individuals with bipolar disorder (BD). However, few prospective studies have examined how CT relates to the longitudinal course of metabolic dysregulation in BD. This study aimed to describe the association between CT exposure and the progression of metabolic disturbances over 36 months in adults with BD.

**Methods:**

In this prospective cohort study, 305 adults diagnosed with BD (Types I and II) were followed across 13 visits over 36 months. Participants were stratified according to CT exposure using the Childhood Trauma Questionnaire—Short Form (CTQ—SF). Metabolic outcomes (HbA1c, BMI, total cholesterol, HDL cholesterol, LDL cholesterol, triglycerides, and hs‐CRP) were analyzed with inverse probability of treatment weighting (IPTW) and linear mixed‐effects models incorporating a group‐by‐time interaction.

**Results:**

Of the 305 participants enrolled, 53 (17.4%) were lost to follow‐up. Longitudinal analyses showed significant CT‐by‐time interactions for all metabolic markers (*p < *0.001). At 36 months, participants exposed to CT showed greater increases than non‐exposed participants in HbA1c (adjusted least‐squares mean difference 1.11%, 95% CI 0.89–1.33), BMI (4.46 kg/m^2^, 95% CI 3.90–5.03), and hs‐CRP (2.68 mg/L, 95% CI 2.52–2.84), together with unfavorable changes in the lipid profile. Effect sizes were large (Cohen's *d* > 0.8). These associations persisted after IPTW weighting for measured baseline covariates.

**Conclusions:**

In this observational cohort, CT exposure was associated with an accelerated trajectory of metabolic deterioration in individuals with BD after weighting for measured baseline characteristics. Because the design is observational and several behavioral, socioeconomic, and illness‐course determinants of metabolic risk were not measured, these findings should be interpreted as associations rather than as causal effects. They nonetheless support the value of early trauma‐informed assessment and of integrated care strategies addressing both psychiatric and physical health in BD. Future research should incorporate detailed lifestyle and treatment‐exposure data, additional inflammatory biomarkers, and preventive interventions to mitigate long‐term cardiometabolic risk in this vulnerable population.

## Graphical abstract

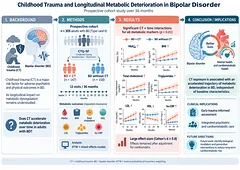

## 1 Introduction

Bipolar disorder (BD) continues to be considered a frequent, persistent, and disabling mental condition. (McIntyre et al. 2020) Over the past several decades, clinical research has attempted to characterize groups at higher risk for BD as well as underlying pathophysiological mechanisms. (Lavin et al. 2022; Kamali et al. 2021; Birmaher et al. 2009; McIntyre et al. 2022) For example, disparate biological markers (e.g., neuroimaging, inflammation, immune, and oxidative stress markers), exhibit differential signatures as a function of illness stage and have been proposed as candidate markers within a multicausal model of the disorder. (Bengesser et al. 2015; Ziani et al. 2022; Scaini et al. 2020)

People with BD are exposed to a higher rate of childhood trauma (CT), adverse childhood adversities including, social adversity, poverty, and malnutrition, when compared to others in the general population. (Rodriguez et al. 2021; Rosenblat et al. 2020; McIntyre et al. 2019) CT is associated with an earlier onset of BD as well as a higher rate of rapid cycling, suicidal ideation and/or behavior, higher rate of hospitalizations when compared to persons without CT. (Galvez‐Florez et al. 2024; Guillen‐Burgos et al. 2023; Guillen‐Burgos et al. 2025)

Multiple physical comorbidities co‐occur at a higher rate in BD than in the general population. These include, but are not limited to, obesity, type 2 diabetes mellitus (T2D), metabolic syndrome (MetS), cardiovascular disease (CVD), and inflammatory bowel disease (IBD). (Foroughi et al. 2022; Liu et al. 2022; McIntyre et al. 2022; Li et al. 2019; Ringin et al. 2023) Not infrequently, metabolic disturbance in BD precedes the late onset of other medical conditions. Likewise, preliminary evidence suggests that metabolic disturbances may also precede the onset of BD after controlling for confounding factors. (Gálvez et al. 2015) Nevertheless, metabolic disturbances driven by obesity can accelerate disease activity, staging, and progression in BD. (Gálvez et al. 2015; McIntyre et al. 2014) Furthermore, metabolic disturbances may enhance loss of functional capacity and risk of developing severe course of illness. (McIntyre et al. 2014; Kemp et al. 2010)

CT has been associated with obesity, metabolic disorders, and CVD in individuals both with and without BD. (Liu et al. 2022) However, few studies have examined how CT influences the course of biomarkers related to metabolic disturbances specifically in BD. (Liu et al. 2022) Evidence suggests that certain subtypes of CT, such as physical abuse, may alter stress‐related biological pathways, contributing to an increased metabolic risk. (Lee et al. 2014) Some findings indicate that CT, in combination with clinical factors such as depressive episodes or seasonality, may predict MetS in specific BD subgroups. (Godin et al. 2021; Osode et al. 2024; Kesebir et al. 2017; Aas et al. 2020) Moreover, CT‐related metabolic vulnerability may precede the onset of medical comorbidities. (McIntyre et al. 2012) Therefore, the main objective of this study was to examine the association between CT exposure and the progression of metabolic outcomes (HbA1c, BMI, total cholesterol, HDL cholesterol, LDL cholesterol, triglycerides, and hs‐CRP) in participants with BD over 36 months of follow‐up.

## 2 Methods

### 2.1 Study Design, Settings, and Population

We performed a 36‐month, longitudinal, prospective observational cohort study. A bipolar disorder cohort has been enrolled at the Center for Clinical and Translational Research in Barranquilla and Bogotá, Colombia, since January 2017. Social, clinical, biological, and cognitive data have been collected at baseline and follow‐up. The study population includes 305 participants with bipolar disorder, followed for 36 months through 13 visits (one visit every three months in addition to the baseline visit). The sample met the following inclusion criteria: (i) ages between 18 and 65 years old; (ii) minimal levels of literacy of participants (reading and writing in Spanish); and (iii) diagnosis of BD I or II according to a Structured Clinical Interview for Diagnostic and Statistical Manual of Mental Disorders—Fifth Edition—Clinician Version (SCID—5—CV). Exclusion criteria were intellectual disability, psychotic‐related disorders, lifetime history of neurological disorders, and bipolar disorder induced by substance/medication or due to another medical condition.

### 2.2 Exposure Variable

CT exposure was assessed with the Childhood Trauma Questionnaire—Short Form (CTQ—SF). CTQ—SF is a self‐administered instrument to assess five subtypes of CT exposure (EA, physical abuse = PA, SA, physical neglect = PN, and emotional neglect = EN) with a 28‐item Likert‐type response (never true = 1, rarely true = 2, sometimes true = 3, often true = 4, and very often true = 5). CTQ—SF exhibited adequate psychometric properties to be included as an objective measurement of CT exposure. The cutoff score (moderate to severe) for each subtype of CT exposure was described in a study to dichotomize each subscore and classify subjects as having or not having a history of CT exposure (≥13 EA; ≥10 PA; ≥8 SA; ≥15 EN; and ≥10 PN). (Bernstein and Fink 1998) Each subscale comprises five items and yields a score ranging from 5 to 25; the CTQ—SF total score (25 clinical items, excluding the three‐item minimization/denial validity scale) ranges from 25 to 125. The Spanish‐language version used in this cohort has shown acceptable internal consistency in Colombian clinical samples (Galvez‐Florez et al. 2024; Guillen‐Burgos et al. 2023; Guillen‐Burgos et al. 2025; Mcintyre et al. 2005). Participants were classified as CT‐exposed when they met or exceeded the moderate‐to‐severe cutoff on at least one subtype. Because dichotomization discards information on trauma severity, two secondary analyses were performed: (i) models in which the CTQ—SF total score was entered as a continuous variable (per 10‐point increment and per standard deviation), and (ii) subtype‐specific models fitted separately for emotional abuse, physical abuse, sexual abuse, emotional neglect and physical neglect. Multiplicity across the five subtypes was addressed with the Benjamini–Hochberg false discovery rate procedure. (McIntyre et al. 2010)

### 2.3 Main Outcome Measures

The outcomes were longitudinal changes in metabolic biomarkers over time, namely HbA1c, BMI, total cholesterol, HDL cholesterol, LDL cholesterol, triglycerides and high‐sensitivity C‐reactive protein (hs‐CRP). Anthropometric measurements and fasting venous blood sampling were repeated at each of the 13 study visits. High‐sensitivity CRP was selected as the inflammatory marker for three reasons. First, it is the inflammatory biomarker with the most widely standardized high‐sensitivity assay and with established clinical cut‐points for cardiovascular risk stratification (<1, 1–3, and >3 mg/L) endorsed by the Centers for Disease Control and Prevention and the American Heart Association. (Fiedorowicz et al. 2008) Second, it is the marker most consistently associated with childhood adversity in meta‐analytic work, which facilitates comparison with the existing literature. (Guo et al. 2006) Third, it was the only inflammatory assay available at both study sites throughout the entire 36‐month follow‐up under routine‐care conditions. Cytokine panels (interleukin‐6, tumor necrosis factor‐α) and adipokines were not available and were therefore not measured; the implications of this restriction are considered in the discussion section as a limitation.

### 2.4 Potential Confounders and Covariables

Variables considered as potential confounders or covariates were age, sex, age at onset of BD, BD subtype, baseline BMI, medical comorbidities (cardiovascular disease, diabetes mellitus, and hyperlipidemia), current psychiatric medication use (antipsychotics and mood stabilizers), previous psychotropic use, and smoking status (current smoker vs. non‐smoker).

Several established determinants of metabolic outcomes were not captured by the cohort protocol and could therefore be included neither in the propensity score nor in the outcome models. These include dietary intake and eating patterns; physical activity and sedentary behavior; alcohol and other substance use; smoking intensity (pack‐years, as distinct from smoking status); sleep duration and quality; educational attainment, income and other indicators of socioeconomic position; access to and utilization of primary medical care; and antihypertensive, lipid‐lowering or glucose‐lowering treatment initiated during follow‐up.

#### 2.4.1 Clinical Course of Bipolar Disorder During Follow‐Up

At every three‐monthly visit, mood state was assessed with a structured clinical evaluation, and affective episodes were recorded prospectively according to DSM‐5‐TR criteria. The following illness‐course variables were collected for the 36‐month period and are reported by CT exposure status in Table S2: number of depressive episodes; number of manic or hypomanic episodes; total time spent symptomatic; predominant polarity; presence of rapid cycling; number of psychiatric hospitalizations; number of suicide attempts; and changes in psychotropic prescription, including initiation or discontinuation of second‐generation antipsychotics, lithium, and valproate. These variables were examined both as descriptors of illness course and, in a secondary model, as time‐varying covariates added to the primary mixed‐effects models, to assess the extent to which the association between CT and metabolic trajectories is explained by the clinical course of the disorder.

### 2.5 Ethics Approval

Clinical data were collected and stored using an electronic data capture system (REDCap). All participants signed written informed consent. The study was approved by the Ethics Committee of Universidad Simon Bolivar and followed the latest version of the Declaration of Helsinki, Belmont Report, Council for International Organizations of Medical Sciences (CIOMS), and Good Clinical Practices.

### 2.6 Statistical Analysis

Statistical analysis was performed using Stata 17MP software (StataCorp LLC, Texas, United States). Relative and absolute frequencies for qualitative variables were calculated as well as a central tendency and dispersion measures for quantitative variables. These analyses were stratified by history of CT exposure in order to identify possible differences in baseline characteristics. For continuous baseline variables, between‐group differences are reported as mean differences with 95% confidence intervals in addition to *p*‐values; categorical variables were compared with the χ^2^ test. Throughout the manuscript, all principal estimates are accompanied by 95% confidence intervals, two‐sided *p*‐values are reported to three decimal places (or as *p < *0.001), and *p < *0.05 was considered statistically significant.

#### 2.6.1 Propensity Score and Inverse Probability of Treatment Weighting Analysis

A propensity score was calculated based on baseline variables including age, sex, age at onset of BD, BD subtype, baseline BMI, medical comorbidities, current psychiatric medication use, previous psychotropic use, and smoking status, considering evidence from the literature suggesting that these factors may be related to history of CT exposure, using a logistic regression model. Potential interactions between some of these variables, including an interaction between sex and medication use, as well as sex and BMI were evaluated to improve the accuracy of the propensity score estimation. Following propensity score estimation, the inverse probability of treatment weighting (IPTW) methodology was used to adjust the comparison between the CT exposure and non‐CT exposure groups by simulating a randomization scenario for each group. The balance between groups was evaluated with balancing tests after IPTW weighting, to obtain a reduction of confounding and selection bias for the selected covariates (Figure S1). Absolute standardized mean difference and covariate balance tests were performed for each covariate, where the reduction in standardized bias was observed for each variable. Balance was considered adequate when the absolute standardized mean difference was below 0.10 for every covariate. Weights were stabilized and trimmed at the first and 99th percentiles to limit the influence of extreme values. It should be emphasized that IPTW can only balance covariates that have been measured; it offers no protection against confounding by the unmeasured variables listed in Section 2.4.

#### 2.6.2 Comparative Analysis

A mixed‐effects model was applied to capture both intra‐ and inter‐individual variability over time and to assess differences in metabolic variables between groups, adjusting for covariates and IPTW weights. To assess differential change over time, we included an interaction between visit and trauma group (CT vs. non‐CT), with adjustment for each participant's baseline measurement. Models included a random intercept for participant and an unstructured residual covariance structure. The analysis was organized following the principles of a target trial emulation framework, in which IPTW weighting and longitudinal modelling are used to approximate the covariate balance that randomization would be expected to produce. (Castilla‐Puentes 2007) We emphasize, however, that this framework reduces confounding only with respect to measured covariates and does not license a causal interpretation of the estimates; all associations reported below are observational. Adjusted regression coefficients for the group‐by‐time interaction (β per 12‐month increment, with 95% confidence intervals) are reported in Table S4.

Marginal effects were calculated using least‐squares means (LS means), together with an estimated effect size (Cohen's *d*) for each visit, to show the magnitude of the difference in change in metabolic biomarkers between the groups compared. All least‐squares mean differences are presented with 95% confidence intervals.

The mixed‐effects models used all available observations under a missing‐at‐random assumption. As a conservative supplementary analysis, missing values were also handled with the last observation carried forward (LOCF) approach, and further sensitivity analyses were conducted using multiple imputation by chained equations (*m* = 20 imputations), with the imputation model including all covariates, all outcomes, and the exposure. In addition, the baseline characteristics of participants who completed the 36‐month follow‐up were compared with those of participants lost to follow‐up, in order to assess the potential for attrition bias (Table S1).

#### 2.6.3 Sensitivity Analysis for Unmeasured Confounding

As residual confounding cannot be excluded in an observational cohort, we quantified how strong an unmeasured confounder would need to be in order to explain away the observed associations. Standardized between‐group differences at 36 months were converted to approximate risk ratios, and the corresponding *E*‐values — for both the point estimate and the limit of the 95% confidence interval closest to the null — were calculated according to the method of VanderWeele and Ding. (Prieto et al. 2014) An *E*‐value represents the minimum strength of association, on the risk‐ratio scale, that an unmeasured confounder would need to have with both CT exposure and the metabolic outcome, conditional on the measured covariates, in order to account fully for the observed association. E‐values are reported in Table S5.

## 3 Results

### 3.1 Demographic Characteristics

The selection of participants is described in Figure 1. A total of 305 participants were analyzed; the mean age was 35.63 years (SD 7.10), with a predominant proportion of participants under 45 years of age (87.87%), and 51.15% were female. Table 1 summarizes the baseline characteristics stratified by history of CT exposure before IPTW analysis.

**FIGURE 1:**
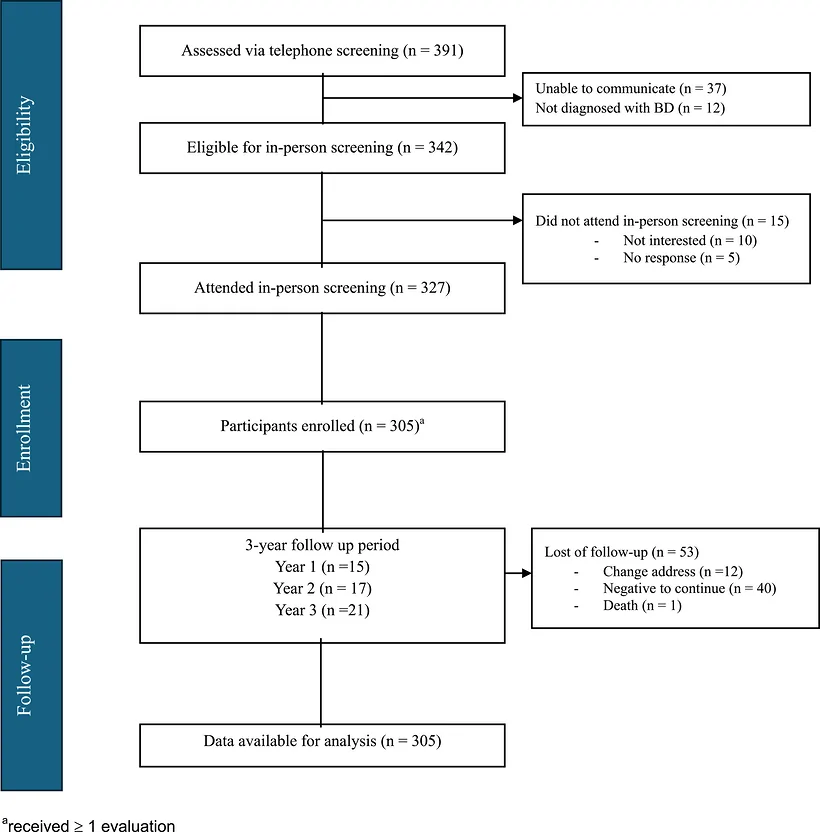
STROBE flowchart of 36‐month of follow‐up.

**TABLE 1:** Baseline characteristics for the cohort of participants.

Characteristic^a^	non‐CT	CT	Total	p‐value^b^
	*n = *126	*n = *179	*n = *305	
Demographic				
Sex				0.74
Female	63 (50.00%)	93 (51.96%)	156 (51.15%)	
Male	63 (50.00%)	86 (48.04%)	149 (48.85%)	
Age	35.75 (7.19)	35.55 (7.05)	35.63 (7.10)	0.82
Age group				0.38
<45	108 (85.71%)	160 (89.39%)	268 (87.87%)	
≥45 to <55	18 (14.29%)	18 (10.06%)	36 (11.80%)	
≥55	0 (0.00%)	1 (0.56%)	1 (0.33%)	
Age at onset	24.93 (4.81)	24.32 (4.67)	24.57 (4.73)	0.27
Clinical				
Bipolar subtype				0.15
Bipolar I	81 (64.29%)	129 (72.07%)	210 (68.85%)	
Bipolar II	45 (35.71%)	50 (27.93%)	95 (31.15%)	
Medication Use				0.69
Antipsychotic	124 (98.41%)	142 (79.32%)	266 (87.21%)	
Mood stabilizers	120 (95.23%)	160 (89.40%)	280 (91.80%)	
Smoking status				0.93
Non‐Smoker	62 (49.21%)	89 (49.72%)	151 (49.51%)	
Smoker	64 (50.79%)	90 (50.28%)	154 (50.49%)	
Previous psychotropic use				0.83
No	60 (47.62%)	83 (46.37%)	143 (46.89%)	
Yes	66 (52.38%)	96 (53.63%)	162 (53.11%)	
BMI classification				0.13
Normal weight	4 (3.17%)	15 (8.38%)	19 (6.23%)	
Overweight	71 (56.35%)	88 (49.16%)	159 (52.13%)	
Obesity	51 (40.48%)	76 (42.46%)	127 (41.64%)	
CVD				0.83
No	121 (96.03%)	171 (95.53%)	292 (95.73%)	
Yes	5 (3.97%)	8 (4.47%)	13 (4.27%)	
Diabetes mellitus				0.63
No	116 (92.07%)	162 (90.50%)	278 (91.15%)	
Yes	10 (7.93%)	17 (9.50%)	27 (8.85%)	
Hyperlipidemia				0.81
No	113 (89.70%)	162 (90.50%)	275 (90.16%)	
Yes	13 (10.30%)	17 (9.50%)	30 (9.84%)	
				

_Abbreviations: BMI, body mass index; CT, childhood trauma; CVD, cardiovascular disorders; non‐CT, non‐childhood trauma._

^a^ _Data are presented as mean (SD) for continuous measures, and n (%) for categorical measures._

^b^ *_p_*_value based on_ *_t_*_‐test or Chi square test._

No statistically significant differences were found between the groups with and without CT exposure in terms of age, sex, or age at onset of BD (*p* > 0.05 in all cases). Baseline medication use is summarized in Table 1. Participants were predominantly diagnosed with BD type I (68.85%), and the use of mood stabilizers was slightly more frequent than that of antipsychotics (91.80% vs. 87.21%). Most participants were in the overweight category (52.13%), followed by obese (41.64%) without a statistically significant difference based on BMI classification. There were no significant differences in smoking status or past psychotropic use between groups (*p* > 0.05). (Table 1).

#### 3.1.1 Attrition and Comparison of Completers With Participants Lost to Follow‐Up

Of the 305 participants enrolled, 53 (17.4%) were lost to follow‐up over the 36‐month period (Year 1, *n = *15; Year 2, *n = *17; Year 3, *n = *21). The reasons recorded were change of address (*n = *12), withdrawal of willingness to continue (*n = *40), and death (*n = *1). All 305 participants contributed at least one post‐baseline assessment and were therefore retained in the mixed‐effects analyses. The baseline characteristics of participants who completed the 36‐month follow‐up (*n = *252) and of those lost to follow‐up (*n = *53) are compared in Table S1.

### 3.2 Baseline Characteristics of Clinical Outcomes

Metabolic outcomes were comparable between the two groups. Table 2 illustrates baseline metabolic ranges, including BMI, HbA1c, glucose, total cholesterol, HDL, LDL, triglycerides, and hs‐CRP, between the groups with and without CT exposure. No statistically or clinically significant differences were observed in any of these parameters between groups (*p* > 0.05 for all outcomes), and all 95% confidence intervals for the between‐group mean differences included the null value.

**TABLE 2:** Primary and secondary baseline outcomes among exposed groups.

Metabolic results^a^	non‐CT	CT	Total	*p*‐value^b^
	*n = *126	*n = *179	*n = *305	
HbA1c (%)	6.89 (1.04)	6.80 (1.07)	6.84 (1.06)	0.44
BMI (kg/m^2^)	29.47 (2.86)	29.43 (3.13)	29.45 (3.02)	0.92
Glucose (mg/dL)	132.82 (14.71)	134.03 (15.56)	133.53 (15.20)	0.50
Total Cholesterol (mg/dL)	196.45 (13.56)	199.00 (17.50)	197.95 (16.01)	0.17
HDL Cholesterol (mg/dL)	45.79 (5.48)	45.50 (4.92)	45.62 (5.15)	0.63
LDL Cholesterol (mg/dL)	115.18 (13.94)	117.88 (14.60)	116.77 (14.37)	0.11
Triglycerides (mg/dL)	159.77 (43.09)	158.39 (39.04)	158.96 (40.70)	0.77
hs‐CRP (mg/L)	3.65 (1.55)	3.55 (1.52)	3.59 (1.53)	0.58

_Abbreviations: BMI, body mass index; CT, childhood trauma; HbA1c, hemoglobin A1c; HDL, high‐density lipoprotein, hs‐CRP, high‐sensitivity C‐reactive protein; LDL, low‐density lipoprotein; non‐CT, non‐childhood trauma._

^a^ _Data are presented as mean (SD) for continuous measures._

^b^ *_p_*_value based on_ *_t_*_‐test test._

With regard to glycemic and lipid indicators, both groups had similar mean levels of HbA1c (non‐CT 6.89% vs. CT 6.80%; mean difference −0.09%, 95% CI −0.33–0.15; *p = *0.44) and of fasting glucose (non‐CT 132.82 mg/dL vs. CT 134.03 mg/dL; mean difference 1.21 mg/dL, 95% CI −2.27–4.69; *p = *0.50). Total, HDL and LDL cholesterol levels were also similar between the groups (total cholesterol mean difference 2.55 mg/dL, 95% CI −1.11–6.21, *p = *0.17; HDL cholesterol −0.29 mg/dL, 95% CI −1.47–0.89, *p = *0.63; LDL cholesterol 2.70 mg/dL, 95% CI −0.58–5.98, *p = *0.11), as were triglycerides (mean difference −1.38 mg/dL, 95% CI −10.71–7.95, *p = *0.77). Levels of high‐sensitivity C‐reactive protein were also similar between groups, averaging 3.59 mg/L (non‐CT 3.65 mg/L vs. CT 3.55 mg/L; mean difference −0.10 mg/L, 95% CI −0.45–0.25; *p = *0.58), indicating that the baseline inflammatory state did not differ appreciably between participants exposed and not exposed to CT.

### 3.3 Analysis of the Effect of Childhood Trauma on Metabolic Impairments

Table 3 and Figure 2 show the comparative analysis by group. The multilevel mixed‐effects models showed that the interaction between CT exposure and time was statistically significant for all metabolic outcomes (*p < *0.001). CT exposure was therefore associated with a more rapid deterioration of metabolic parameters over time than was observed in participants without CT exposure. At 36 months, the adjusted least‐squares mean differences (CT minus non‐CT) were 1.11% (95% CI 0.89 to 1.33) for HbA1c, 4.46 kg/m^2^ (95% CI 3.90–5.03) for BMI, 37.67 mg/dL (95% CI 34.89–40.45) for total cholesterol, −5.63 mg/dL (95% CI −6.88 to −4.39) for HDL cholesterol, 30.18 mg/dL (95% CI 27.44–32.92) for LDL cholesterol, 33.72 mg/dL (95% CI 25.29–42.16) for triglycerides and 2.68 mg/L (95% CI 2.52–2.84) for hs‐CRP. Marginal effect estimates with LS Means, together with effect sizes (Cohen's d) for each measurement, also demonstrated statistically significant greater metabolic disturbances in BD participants with CT exposure versus non‐CT‐exposed with regard to changes in HbA1c, BMI, cholesterol, and triglycerides levels as well as hs‐CRP values, with a Cohen *d* effect size greater than 0.8, corresponding to large effect sizes (Table 3, Figure 2). Adjusted regression coefficients for the group‐by‐time interaction are reported in Table S4.

**TABLE 3:** Summary of comparative analysis.

End point^a^	Group	Baseline mean (SD)	At 36 months, change from baseline, LS mean (SE)	Between‐group difference at 36 months		
				LS mean difference (95% CI)	*p* value	Cohen *d* effect size
HbA1c (%)	non‐CT	6.89 (1.04)	−0.18 (0.08)	Difference: 1.11 (0.89–1.33)	< 0.001	1.11
	CT	6.80 (1.07)	0.93 (0.08)			
BMI (kg/m2)	non‐CT	29.47 (2.86)	0.87 (0.20)	Difference: 4.46 (3.90–5.03)	< 0.001	1.86
	CT	29.43 (3.13)	5.34 (0.20)			
Total cholesterol (mg/dL)	non‐CT	196.45 (13.56)	−22.09 (0.94)	Difference: 37.67 (34.89–40.45)	< 0.001	3.17
	CT	199.00 (17.50)	15.57 (1.05)			
HDL cholesterol (mg/dL)	non‐CT	45.79 (5.48)	−0.89 (0.52)	Difference: ‐5.63 (‐6.88 to ‐4.39)	< 0.001	1.21
	CT	45.50 (4.92)	−6.52 (0.36)			
LDL cholesterol (mg/dL)	non‐CT	115.18 (13.94)	−24.29 (0.93)	Difference: 30.18 (27.44–32.92)	< 0.001	2.51
	CT	117.88 (14.60)	5.90 (1.03)			
Triglycerides (mg/dL)	non‐CT	159.77 (43.09)	−20.51 (3.23)	Difference: 33.72 (25.29–42.16)	< 0.001	0.97
	CT	158.39 (39.04)	13.21 (2.83)			
hs‐CRP (mg/L)	non‐CT	3.65 (1.55)	−1.35 (0.04)	Difference: 2.68 (2.52–2.84)	< 0.001	4.03
	CT	3.55 (1.52)	1.32 (0.07)			

_Abbreviations: BMI: body mass index; CT: childhood trauma; HbA1c: hemoglobin A1c; HDL: high‐density lipoprotein; hs‐CRP: high‐sensitivity C‐reactive protein; LDL: low‐density lipoprotein; non‐CT: non‐childhood trauma._

^a^ _Estimates are based on a mixed model for repeated measures with inverse probability of treatment weighting; LS mean differences are presented with 95% confidence intervals._

**FIGURE 2:**
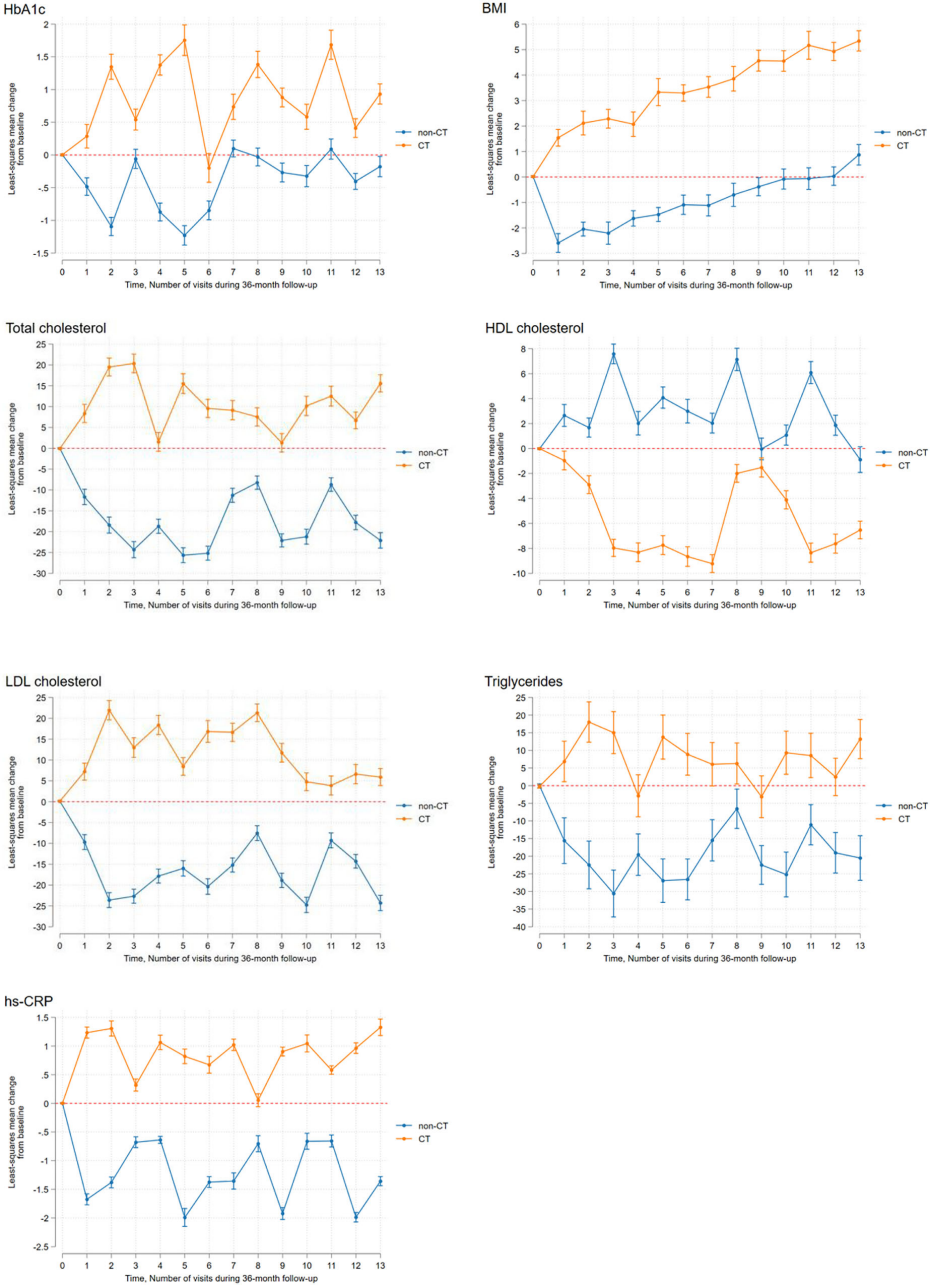
Change from baseline in metabolic outcomes.

### 3.4 Childhood Trauma Severity and Subtype‐Specific Analyses

Mean CTQ—SF total scores were 58.4 (SD 13.2) in the CT‐exposed group and 33.1 (SD 5.8) in the non‐exposed group (mean difference 25.3, 95% CI 22.9–27.7). Subscale scores for emotional abuse, physical abuse, sexual abuse, emotional neglect and physical neglect are reported in Table S3. When the CTQ—SF total score was modelled as a continuous variable, each 10‐point increment was associated with a difference at 36 months of β = 0.19% (95% CI 0.14–0.24) in HbA1c, β = 0.71 kg/m^2^ (95% CI 0.58–0.84) in BMI and β = 0.44 mg/L (95% CI 0.38–0.50) in hs‐CRP. In subtype‐specific models, physical abuse showed the strongest associations (HbA1c β = 0.26, 95% CI 0.18–0.34; BMI β = 0.83, 95% CI 0.63–1.03; hs‐CRP β = 0.52, 95% CI 0.43–0.61; all *q* < 0.001), followed by emotional abuse and emotional neglect (both *q* < 0.001), whereas sexual abuse (*q* = 0.021) and physical neglect (*q* = 0.048) showed weaker associations (Table S3). Associations were directionally consistent across all five domains but were concentrated in the abuse rather than the neglect domains, and a graded increase across CTQ—SF severity tertiles was apparent for all three principal outcomes.

### 3.5 Clinical Course of Bipolar Disorder During Follow‐Up

Over the 36‐month follow‐up, participants experienced a mean of 3.8 mood episodes (2.4 depressive and 1.3 manic or hypomanic), and 73 (23.9%) required at least one psychiatric hospitalization. Predominant polarity was depressive in 170 (55.7%) and manic in 135 (44.3%); 52 (17.0%) met criteria for rapid cycling; and 36 (11.8%) recorded at least one suicide attempt. Cumulative exposure to second‐generation antipsychotics, expressed as olanzapine‐equivalent dose‐years, was 21.6 (SD 10.7) in the CT‐exposed group and 18.2 (SD 9.4) in the non‐exposed group. These variables are compared by CT exposure status in Table S2. When the illness‐course variables that differed between groups were added as time‐varying covariates to the mixed‐effects models, the CT‐by‐time interaction remained significant but attenuated, with adjusted least‐squares mean differences at 36 months of 0.84% (95% CI 0.61–1.07) for HbA1c, 3.61 kg/m^2^ (95% CI 3.02–4.20) for BMI and 2.21 mg/L (95% CI 2.03–2.39) for hs‐CRP.

### 3.6 Sensitivity Analyses

Estimates were materially unchanged in the multiple‐imputation analysis relative to the LOCF analysis presented above (HbA1c 1.09%, 95% CI 0.86–1.32; BMI 4.39 kg/m^2^, 95% CI 3.82–4.96; hs‐CRP 2.64 mg/L, 95% CI 2.47–2.81). E‐values for the observed associations and for the confidence limits closest to the null are presented in Table S5 (HbA1c 4.9 and 3.9 for the confidence limit closest to the null; BMI 10.3 and 8.3; hs‐CRP 77.8 and 62.4). For HbA1c, an unmeasured confounder would need to be associated with both CT exposure and the outcome by a risk ratio of at least 4.9, over and above the measured covariates, to account fully for the observed association. Associations of that magnitude are not implausible for behavioral and socioeconomic variables, which is why the absence of dietary, physical‐activity, and socioeconomic data is treated here as a principal limitation.

## 4 Discussion

In this 36‐month longitudinal cohort study of people with BD, we found that exposure to CT was significantly associated with a higher incidence and greater severity of metabolic disturbances over time, with these disturbances becoming more prevalent and pronounced throughout the follow‐up period. Despite no significant baseline differences in metabolic parameters or clinical characteristics between CT‐exposed and non‐exposed groups, participants with a history of CT demonstrated a marked and sustained worsening in metabolic biomarkers, including HbA1c, BMI, lipid profiles, triglycerides, and hs‐CRP levels. The effect sizes were consistently large, with the most pronounced differences observed in inflammatory (hs‐CRP) and glycemic (HbA1c) markers. These findings are consistent with CT exposure acting as a marker of—and potentially a contributor to—metabolic vulnerability in BD, and they underscore the need for early identification and longitudinal monitoring of physical health outcomes in this high‐risk subgroup. Because the design is observational, they do not establish that CT causes metabolic deterioration.

The trajectory of HbA1c in our CT‐exposed group aligns with existing literature highlighting the metabolic vulnerability of patients with BD, particularly with regard to glucose metabolism. Previous studies have consistently demonstrated that individuals with BD frequently exhibit insulin resistance and impaired glucose tolerance, often resulting in elevated HbA1c levels and a higher likelihood of developing type 2 diabetes mellitus. (Goldstein et al. 2015; SayuriYamagata et al. 2017; Fiedorowicz et al. 2008) Consistent with these reports, we observed a significant increase in HbA1c over time among CT‐exposed participants, in contrast to the modest decline seen in non‐exposed individuals. This divergence may be partially attributed to the metabolic side effects of psychotropic medications—particularly atypical antipsychotics—which are known to elevate glucose and HbA1c levels. (Tao et al. 2022; Huang et al. 2018) Although the presence or absence of psychotropic drug classes at baseline was incorporated into the propensity score, cumulative exposure, dose, and within‐class agent were not quantified, and baseline use of antipsychotics was not identically distributed across the two groups (Table 1). Differential pharmacological exposure therefore remains a plausible partial explanation for the divergent HbA1c trajectories that we observed. It is also possible that CT exposure confers a distinct vulnerability to these metabolic effects through underlying biological mechanisms, including stress‐mediated dysregulation of the hypothalamic–pituitary–adrenal (HPA) axis and impaired insulin signaling; the present data cannot distinguish between these accounts.

Moreover, the observed deterioration in metabolic parameters in CT‐exposed individuals underscores the complex interplay between early‐life adversity, chronic stress exposure, and physical health outcomes in BD. A growing body of research has linked CT and cumulative psychosocial stressors to altered metabolic functioning, including heightened inflammatory responses and poor glycemic control. (Aldinger and Schulze 2017) Our study extends these findings by demonstrating that CT exposure not only influences psychiatric course and severity but also accelerates metabolic decline over time, even after adjusting for potential confounders. One interpretation is that CT acts as a long‐term biological sensitizer, amplifying the metabolic burden of BD and increasing the risk of serious comorbidities such as cardiovascular disease and metabolic syndrome; this interpretation remains hypothetical and cannot be tested with the present data. The association may nonetheless help to characterize a more severe course of illness. Consequently, we suggest that adults with BD be routinely evaluated for a history of CT, given its association with bipolar presentation and with metabolic risk. (McIntyre et al. 2022)

In line with previous reports, our study identified significantly greater increases in total cholesterol, HDL/LDL cholesterol, and triglyceride levels over time among BD participants with a history of CT. Dyslipidemia is well‐documented in individuals with BD, with multiple studies demonstrating elevated prevalence of obesity, metabolic syndrome, and adverse lipid profiles in this population. (SayuriYamagata et al. 2017; Etain and Aas 2021; Zhang et al. 2023; Ringin et al. 2024) A recent systematic review emphasized the metabolic burden among BD patients, particularly those prescribed atypical antipsychotics, highlighting the increased risk of elevated triglycerides and total cholesterol (Tao et al. 2022).

Importantly, while both groups in our study had comparable lipid levels at baseline, the CT‐exposed group exhibited a trajectory characterized by significant longitudinal increases, which is compatible with, though by no means proof of, an effect of early‐life trauma on dyslipidemic progression; medication effects and health behaviors were not measured in sufficient detail to be excluded as explanations. These findings highlight the importance of monitoring lipid metabolism longitudinally in BD patients, especially in those with a known trauma history.

Furthermore, our findings resonate with emerging evidence that mood states in BD can influence lipid metabolism. Huang et al. reported that BD patients in depressive states presented with higher triglyceride levels, whereas those in manic states demonstrated the lowest rates of hypertriglyceridemia (Huang et al. 2018). Given that CT exposure is associated with greater depressive burden, increased chronicity, and more frequent affective episodes, it is plausible that the elevated triglyceride levels observed in the CT‐exposed group in our cohort may, in part, reflect the downstream physiological impact of sustained or recurrent depressive episodes. This mood‐dependent metabolic variation further reinforces the need to integrate psychiatric staging and affective symptom monitoring into cardiometabolic risk assessment. The combination of trauma exposure, mood instability, and psychotropic side effects likely constitutes a “metabolic triple hit,” compounding the long‐term risk of cardiovascular disease in BD.

Our study also demonstrated a substantial increase in BMI over time among BD participants with a history of CT, in contrast to only modest weight changes in the non‐exposed group. This observation is consistent with the broader literature showing a strong association between CT exposure and obesity‐related outcomes in individuals with BD. Prior research indicates that nearly half of individuals with BD report a history of CT, which has been linked not only to earlier illness onset and more severe affective symptomatology but also to greater metabolic burden, including elevated BMI. (Galvez‐Florez et al. 2024; Guillen‐Burgos et al. 2023; Guillen‐Burgos et al. 2025; Aldinger and Schulze 2017; Etain and Aas 2021)

The mechanisms underlying this association are likely multifactorial, involving trauma‐related dysregulation of the HPA axis, heightened inflammatory tone, and behavioral sequelae such as sedentary behavior or substance use. The longitudinal rise in BMI observed in our CT‐exposed cohort supports the notion that early‐life trauma contributes to enduring disruptions in energy regulation and metabolic homeostasis, thereby increasing the risk of developing full metabolic syndrome. These findings underscore the need for integrated interventions that simultaneously address psychological trauma and physical health vulnerabilities in individuals with BD.

Finally, our findings of elevated hs‐CRP levels over time in CT‐exposed individuals with BD are further supported by meta‐analytic evidence demonstrating that CT is consistently associated with a pro‐inflammatory phenotype in adulthood. A recent meta‐analysis including over 16,000 individuals found that exposure to early‐life adversity was significantly associated with increased peripheral levels of inflammatory markers, including C‐reactive protein (CRP), interleukin‐6 (IL‐6), and tumor necrosis factor‐alpha (TNF‐α). Importantly, the strongest associations between CT and CRP were observed in clinical psychiatric populations, suggesting that inflammation may represent a key biological mediator linking early trauma to poor physical and mental health outcomes. (McIntyre et al. 2020) These findings reinforce the biological plausibility of our results, indicating that systemic inflammation—potentially shaped by specific trauma subtypes—may underlie the accelerated metabolic deterioration observed in BD patients with a history of trauma. This growing body of evidence positions inflammation not only as a biomarker of disease burden, but also as a promising therapeutic target in trauma‐exposed individuals with BD.

Several non‐biological explanations for these findings deserve equal consideration, and the present design cannot adjudicate between them. First, pharmacological exposure is the most immediate alternative account: second‐generation antipsychotics, valproate and, to a lesser degree, lithium produce weight gain, insulin resistance and dyslipidaemia, and neither cumulative dose nor the specific within‐class agent was quantified in this cohort. Second, health behaviors that cluster with early adversity—energy‐dense diet, physical inactivity, disrupted sleep, heavier smoking, and higher alcohol consumption—are established mediators of the association between childhood maltreatment and adult cardiometabolic disease, and none of them was measured here; smoking, in particular, was recorded only as a binary status rather than as intensity or pack‐years. Third, socioeconomic adversity is a common antecedent of both childhood maltreatment and adult metabolic disease, so that, in the absence of data on education, income or material deprivation, part of the association we report may reflect confounding by socioeconomic position rather than any effect of trauma itself. Fourth, differential access to and engagement with medical care could generate the same pattern: participants with a trauma history may be less likely to be prescribed statins, metformin or antihypertensive agents and more likely to disengage from routine monitoring, which would produce worsening biomarker trajectories without any direct biological pathway. Fifth, obesity‐related behaviors and inflammation are themselves associated with depressive recurrence, which in turn worsens health behavior, so bidirectional and reverse pathways are plausible. Finally, the retrospective assessment of childhood trauma is susceptible to recall bias that may vary with current mood state, which could inflate the observed association. These explanations are not mutually exclusive with the neuroendocrine and inflammatory mechanisms outlined above; disentangling them will require cohorts in which lifestyle, socioeconomic, and treatment‐exposure data are collected prospectively and in detail.

Taken together, our findings emphasize the urgent need for integrated clinical approaches that consider both psychiatric and metabolic dimensions of care in individuals with BD, particularly those with a history of CT. The robust and consistent associations between CT exposure and adverse metabolic trajectories—spanning glycemic control, lipid profiles, BMI, and systemic inflammation—suggest that trauma‐informed assessment should be a standard component of routine care in BD. Identifying history of early trauma may allow clinicians to implement targeted preventive strategies such as dietary counseling, physical activity promotion, regular metabolic screening, and early pharmacologic interventions to mitigate long‐term cardiometabolic risk. Additionally, psychotherapeutic modalities that address trauma‐related stress and emotion regulation (e.g., trauma‐focused CBT, EMDR) could have downstream benefits for physical health by potentially modulating inflammatory and neuroendocrine dysregulation. As such, a transdiagnostic, trauma‐sensitive, and multidisciplinary approach is essential for optimizing both mental and physical health outcomes in this vulnerable population.

Despite the strengths of our study—a well‐characterized longitudinal cohort with 13 repeated assessments over 36 months, propensity‐score weighting, and comprehensive metabolic profiling—several limitations must be emphasized. First, the design is observational. Although IPTW balanced the measured covariates and the analysis was framed within a target trial emulation approach, no weighting scheme can address confounding by variables that were never measured, and the estimates reported here should be read as associations rather than causal effects. Second, and most importantly, several established determinants of metabolic outcomes were not captured: diet, physical activity, alcohol and substance use, smoking intensity, sleep, socioeconomic position, access to medical care, and the cumulative dose and duration of individual psychotropic agents. Residual confounding by these variables is likely, and the E‐values reported in Table S5 should be interpreted with that in mind. Third, the CTQ—SF is a retrospective self‐report instrument and is therefore subject to recall bias, which may itself be influenced by current mood state; although we now report severity scores and subtype‐specific analyses, the dichotomization used in the primary analysis inevitably discards information on trauma severity and chronicity, and age at trauma exposure was not recorded. Fourth, 53 participants (17.4%) were lost to follow‐up. Although completers and non‐completers were compared (Table S1) and multiple imputation produced consistent results, attrition bias cannot be excluded, and the LOCF approach used in the supplementary analysis is conservative but may understate within‐person variability. Fifth, hs‐CRP was the only inflammatory biomarker available. It is a non‐specific hepatic acute‐phase reactant that is strongly influenced by adiposity, and in the absence of IL‐6, TNF‐α, or other cytokines, we cannot determine whether the inflammatory signal we observed is independent of the concurrent increase in BMI or secondary to it; the inflammatory mechanism we invoke therefore remains inferential. Sixth, the magnitude of the between‐group differences we observed is large relative to that reported in comparable cohorts, and independent replication is required before these estimates are used for risk prediction or clinical decision‐making. Finally, the cohort was recruited at two specialist centers in Colombia, so generalizability to other health systems, ethnic groups, and care settings is uncertain. Future studies should incorporate prospective assessment of trauma subtype and severity, a broader inflammatory panel (e.g. IL‐6, TNF‐α), objective measures of diet and physical activity, cumulative psychotropic exposure and mood variability, in larger and more diverse samples, in order to explore potential mediators and moderators—such as genetic vulnerability, neuroendocrine dysregulation and resilience factors—that could inform personalized interventions.

## 5 Conclusions

In conclusion, our findings provide longitudinal evidence of an association between CT and a more severe progression of metabolic disturbances in individuals with BD. The observed trajectories of worsening glycemic control, dyslipidemia, elevated BMI, and heightened inflammation in CT‐exposed individuals underscore the enduring impact of early‐life adversity on physical health outcomes. These results highlight the importance of incorporating trauma screening into routine psychiatric evaluations and adopting integrated, multidisciplinary strategies that address both mental and metabolic health. By identifying high‐risk subgroups and intervening early, clinicians may be able to mitigate the compounding burden of metabolic comorbidities and improve long‐term outcomes for individuals living with BD. Given the observational design and the behavioral, socioeconomic, and treatment‐related factors that were not measured, these findings should be regarded as hypothesis‐generating and require replication in cohorts with detailed lifestyle and treatment‐exposure data. In addition, future research should evaluate whether progressive changes in metabolic function mediate changes in cognition in BD, and the extent to which metabolic disturbances are additive to, and independent of, CT with respect to cognitive outcomes. (Ringin et al. 2023; Ringin et al. 2024; Zhang et al. 2023)

## Author Contributions

**Juan F. Galvez‐Florez**: conceptualization, data curation, formal analysis, investigation, writing – original draft, writing – review and editing. **Hernán F. Guillen‐Burgos**: conceptualization, data curation, formal analysis, investigation, writing – original draft, writing – review and editing. **Sergio Moreno‐López**: data curation, formal analysis, methodology. **Mariana Ramírez‐Posada**: writing – original draft, writing – review and editing. **Juan‐Manuel Anaya**: writing – original draft, writing – review and editing. **Roger S. McIntyre**: conceptualization, investigation, supervision, writing – original draft, writing – review and editing.

## AI disclosure statement

During the preparation of this work, the author(s) used Gemini from Google to generate/enhance the graphical schematic abstract. After using this tool, the author(s) reviewed and edited the image to ensure accuracy and take full responsibility for the content of the published figure.

## Funding

This study was supported by Center for Clinical and Translational Research, Pontificia Universidad Javeriana, and Universidad Simon Bolivar. The funders had no role in the study design, the data collection, types of analyses established, the decision to publish results, or the preparation of the manuscript.

## Conflicts of Interest

Dr. Juan F. Galvez‐Florez is also CEO of the Latin‐American Society of Consultation‐Liaison Psychiatry in Bogota, Colombia. Additionally, he works as a psychiatrist and researcher at the Center for Clinical and Translational Research at Clinica de Nuestra Señora de la Paz in Bogota Colombia. Dr. Hernán F. Guillen‐Burgos has received research grant support from the Ministry of Science, Technology, and Innovation (MinCiencias) in Bogota, Colombia, and United Kingdom Research & Innovation Unit; speaker fees from Roche, Pfizer, Abbott, and GSK. Dr Guillen‐Burgos also works at the Center for Clinical and Translational Research at Clinica de Nuestra Señora de la Paz, and Department of Psychiatry of Mental Health at Pontificia Universidad Javeriana/Hospital Universitario San Ignacio in Bogota, Colombia Dr. Roger S. McIntyre has received research grant support from CIHR/GACD/National Natural Science Foundation of China (NSFC) and the Milken Institute; speaker/consultation fees from Lundbeck, Janssen, Alkermes, Neumora Therapeutics, Boehringer Ingelheim, Sage, Biogen, Mitsubishi Tanabe, Purdue, Pfizer, Otsuka, Takeda, Neurocrine, Neurawell, Sunovion, Bausch Health, Axsome, Novo Nordisk, Kris, Sanofi, Eisai, Intra‐Cellular, NewBridge Pharmaceuticals, Viatris, Abbvie and Atai Life Sciences. The remaining authors declare no conflicts of interest.

## Supporting information




**Figure S1**: Covariate balance before and after inverse probability of treatment weighting (absolute standardized mean differences).
**Table S1**: Baseline characteristics of participants who completed the 36‐month follow‐up (*n = *252) compared with participants lost to follow‐up (*n = *53).
**Table S2**: Clinical course of bipolar disorder over 36 months by childhood trauma exposure status (mood episodes, predominant polarity, rapid cycling, hospitalizations, suicide attempts, and changes in psychotropic prescription).
**Table S3**: CTQ—SF total and subscale scores by group, and subtype‐specific associations between childhood trauma domains, and metabolic outcomes at 36 months (adjusted β, 95% CI, Benjamini–Hochberg‐adjusted *p* values).
**Table S4**: Adjusted regression coefficients for the group‐by‐time interaction from the mixed‐effects models (β per 12‐month increment, 95% CI).
**Table S5**: E‐values for the association between childhood trauma exposure and each metabolic outcome at 36 months.

## Data Availability

The data that support the findings of this study are available on request from the corresponding author. The data are not publicly available due to privacy or ethical restrictions
