# Supplementary material for: Childhood Trauma Exposure Associated With a More Severe Course of Metabolic Disturbances in Bipolar Disorder: A 36‐Month Longitudinal Prospective Cohort Study
Source: Brain Behav. 2026 Oct 24;16(10):e71789. doi: 10.1002/brb3.71789 (PMC13695791; doi:10.1002/brb3.71789)
Supplement: Supplementary file 1 [file BRB3-16-e71789-s001.docx]

**Supplementary Material**

**Figure S1: Complementary information IPTW analysis: Balance before and after IPTW analysis**

Figure S1. Covariate balance before and after inverse probability of treatment weighting (absolute standardised mean differences).

**Table S1. Completers vs. participants lost to follow-up**

| **Baseline characteristic** | **Completers (n = 252)** | **Lost to follow-up (n = 53)** | **Difference (95% CI)** | **p** |
| --- | --- | --- | --- | --- |
| Age, years, mean (SD) | 35.4 (7.0) | 36.7 (7.5) | 1.3 (−0.8 to 3.4) | 0.22 |
| Female, n (%) | 131 (52.0) | 25 (47.2) | — | 0.52 |
| Bipolar I, n (%) | 171 (67.9) | 39 (73.6) | — | 0.41 |
| CT-exposed, n (%) | 146 (57.9) | 33 (62.3) | — | 0.55 |
| Age at onset, years, mean (SD) | 24.6 (4.7) | 24.3 (4.9) | −0.3 (−1.7 to 1.1) | 0.68 |
| BMI, kg/m², mean (SD) | 29.4 (3.0) | 29.7 (3.2) | 0.3 (−0.6 to 1.2) | 0.51 |
| HbA1c, %, mean (SD) | 6.83 (1.05) | 6.90 (1.11) | 0.07 (−0.24 to 0.38) | 0.66 |
| hs-CRP, mg/L, mean (SD) | 3.57 (1.52) | 3.68 (1.58) | 0.11 (−0.34 to 0.56) | 0.63 |
| Current smoker, n (%) | 125 (49.6) | 29 (54.7) | — | 0.50 |

Table S1. Baseline characteristics of participants who completed the 36-month follow-up (n = 252) compared with participants lost to follow-up (n = 53).

**Table S2. Clinical course over 36 months by CT exposure**

| **Illness-course variable** | **non-CT (n = 126)** | **CT (n = 179)** | **Difference (95% CI)** | **p** |
| --- | --- | --- | --- | --- |
| Depressive episodes, mean (SD) | 1.9 (1.3) | 2.8 (1.6) | 0.9 (0.6 to 1.2) | <0.001 |
| Manic/hypomanic episodes, mean (SD) | 1.2 (1.0) | 1.4 (1.2) | 0.2 (−0.1 to 0.5) | 0.14 |
| Weeks symptomatic, mean (SD) | 28.4 (14.1) | 39.6 (17.8) | 11.2 (7.5 to 14.9) | <0.001 |
| Predominant polarity depressive, n (%) | 58 (46.0) | 112 (62.6) | — | 0.004 |
| Rapid cycling, n (%) | 14 (11.1) | 38 (21.2) | — | 0.019 |
| ≥1 psychiatric hospitalisation, n (%) | 21 (16.7) | 52 (29.1) | — | 0.012 |
| ≥1 suicide attempt, n (%) | 9 (7.1) | 27 (15.1) | — | 0.033 |
| SGA started during follow-up, n (%) | 30 (23.8) | 61 (34.1) | — | 0.055 |
| Olanzapine-equivalent dose-years, mean (SD) | 18.2 (9.4) | 21.6 (10.7) | 3.4 (1.0 to 5.8) | 0.006 |

Table S2. Clinical course of bipolar disorder over 36 months by childhood trauma exposure status (mood episodes, predominant polarity, rapid cycling, hospitalizations, suicide attempts and changes in psychotropic prescription).

**Table S3. CTQ-SF scores and subtype-specific associations**

| **CTQ-SF domain** | **non-CT, mean (SD)** | **CT, mean (SD)** | **β HbA1c (95% CI)** | **β BMI (95% CI)** | **β hs-CRP (95% CI)** | **q** |
| --- | --- | --- | --- | --- | --- | --- |
| Total score (25–125) | 33.1 (5.8) | 58.4 (13.2) | 0.19 (0.14 to 0.24) | 0.71 (0.58 to 0.84) | 0.44 (0.38 to 0.50) | <0.001 |
| Emotional abuse | 7.2 (2.1) | 13.8 (4.0) | 0.21 (0.13 to 0.29) | 0.68 (0.49 to 0.87) | 0.46 (0.37 to 0.55) | <0.001 |
| Physical abuse | 6.1 (1.6) | 11.4 (3.5) | 0.26 (0.18 to 0.34) | 0.83 (0.63 to 1.03) | 0.52 (0.43 to 0.61) | <0.001 |
| Sexual abuse | 5.6 (1.2) | 9.7 (3.8) | 0.11 (0.02 to 0.20) | 0.34 (0.12 to 0.56) | 0.19 (0.09 to 0.29) | 0.021 |
| Emotional neglect | 8.4 (2.6) | 15.9 (4.4) | 0.18 (0.10 to 0.26) | 0.62 (0.43 to 0.81) | 0.41 (0.32 to 0.50) | <0.001 |
| Physical neglect | 6.8 (2.0) | 11.2 (3.6) | 0.09 (0.00 to 0.18) | 0.29 (0.07 to 0.51) | 0.15 (0.05 to 0.25) | 0.048 |

Table S3. CTQ-SF total and subscale scores by group, and subtype-specific associations between childhood trauma domains and metabolic outcomes at 36 months (adjusted β, 95% CI, Benjamini–Hochberg-adjusted p values).

**Table S4. Adjusted group-by-time interaction coefficients**

| **Outcome** | **β per 12 months (95% CI)** | **p** |
| --- | --- | --- |
| HbA1c, % | 0.37 (0.30 to 0.44) | <0.001 |
| BMI, kg/m² | 1.49 (1.30 to 1.68) | <0.001 |
| Total cholesterol, mg/dL | 12.56 (11.63 to 13.49) | <0.001 |
| HDL cholesterol, mg/dL | −1.88 (−2.29 to −1.47) | <0.001 |
| LDL cholesterol, mg/dL | 10.06 (9.15 to 10.97) | <0.001 |
| Triglycerides, mg/dL | 11.24 (8.43 to 14.05) | <0.001 |
| hs-CRP, mg/L | 0.89 (0.84 to 0.94) | <0.001 |

Table S4. Adjusted regression coefficients for the group-by-time interaction from the mixed-effects models (β per 12-month increment, 95% CI).

**Table S5. E-values**

| **Outcome** | **Cohen's d (Table 3)** | **Approx. RR** | **E-value (estimate)** | **E-value (CI limit)** |
| --- | --- | --- | --- | --- |
| HbA1c | 1.11 | 2.7 | 4.9 | 3.9 |
| BMI | 1.86 | 5.4 | 10.3 | 8.3 |
| Total cholesterol | 3.17 | 17.9 | 35.3 | 28.4 |
| HDL cholesterol | 1.21 | 3.0 | 5.5 | 4.2 |
| LDL cholesterol | 2.51 | 9.8 | 19.1 | 15.4 |
| Triglycerides | 0.97 | 2.4 | 4.3 | 3.3 |
| hs-CRP | 4.03 | 39.1 | 77.8 | 62.4 |

Table S5. E-values for the association between childhood trauma exposure and each metabolic outcome at 36 months, for the point estimate and for the confidence limit closest to the null.
